# Medication persistence for psoriatic arthritis in a Brazilian real-world setting

**DOI:** 10.4155/fsoa-2018-0101

**Published:** 2019-01-18

**Authors:** Michael Ruberson Ribeiro da Silva, Jéssica Barreto Ribeiro dos Santos, Alessandra Maciel Almeida, Alexander Itria, Adriana Maria Kakehasi, Juliana Alvares Teodoro, Francisco de Assis Acurcio

**Affiliations:** 1 Department of Social Pharmacy, Postgraduate Program in Medicines & Pharmaceutical Assistance, College of Pharmacy, Federal University of Minas Gerais, President Antônio Carlos Avenue, 6627, Campus Pampulha, Belo Horizonte, Minas Gerais 31270–901, Brazil; 2 Department of Social Pharmacy, College of Pharmacy, Federal University of Minas Gerais, President Antônio Carlos Avenue, 6627, Campus Pampulha, Belo Horizonte, Minas Gerais 31270–901, Brazil; 3 Department of Public Health, Institute of Tropical Pathology & Public Health, Federal University of Goiás, 235 Street, Eastern University Sector, Goiânia, Goias, 74605-050, Brazil; 4 Department of Musculoskeletal System, Medicine School, Federal University of Minas Gerais, Professor Alfredo Balena Avenue, 190, Belo Horizonte, Minas Gerais, 30130-100, Brazil; 5 Department of Preventive and Social Medicine, Medicine School, Federal University of Minas Gerais, Professor Alfredo Balena Avenue, 190, Belo Horizonte, Minas Gerais, 30130-100, Brazil

**Keywords:** antirheumatic agents, medication persistence, psoriatic arthritis

## Abstract

**Aim:**

To evaluate the persistence of biological (TNF inhibitor [anti-TNF]) and synthetic (conventional synthetic disease-modifying antirheumatic drugs [csDMARDs]) antirheumatic agents for psoriatic arthritis and their associated factors.

**Methods:**

A historical cohort was developed. Persistence and associated factors were evaluated at 6 and 12 months.

**Results:**

A total of 161 patients were included. The anti-TNF treatment presented higher persistence as compared with csDMARDs at 6 (83.4 vs 50.8%; p < 0.05) and 12 months (66.4 vs 35.6%; p < 0.05). From anti-TNFs, adalimumab and etanercept presented similar persistence, along with leflunomide and methotrexate among the csDMARDs. The factors associated with non-persistence with regard to anti-TNF agents were female sex and use of infliximab.

**Conclusion:**

Anti-TNF agents are important therapeutic alternatives and present lower rates of discontinuation as compared with csDMARDs.

## Lay abstract

Conventional synthetic disease-modifying antirheumatic drugs (csDMARD) and anti-TNF agents were included in the Brazilian National Health System in 2009, yet were not previously the target of evaluations in a Brazilian context. Persistence, that is the time between starting and discontinuing the drug, is a proxy of effectiveness and safety for antirheumatic agents. This study evaluated medication persistence and relevant factors associated with it. Patients using infliximab showed a lower medication persistence when compared with adalimumab and etanercept. The medication persistence with anti-TNF agents was higher when compared with csDMARDs. Female sex and infliximab were predictors of medication non-persistence.

Psoriatic arthritis (PsA) is a chronic inflammatory arthritis that affects axial and peripheral joints, entheses, tendons and fascias; and is associated with cutaneous psoriasis [1,2]. It was described for the first time in 1956 by Wright and it is considered the most common extracutaneous manifestation of psoriasis [1,3].

PsA causes a decrease in occupational function and psychosocial morbidity. When compared with the general population, PsA patients present a decrease in quality of life (QoL), a functional impairment, psychosocial inability and a significant increase in their mortality index [4,5]. This impact on QoL is related to pain, skin problems, functional disability and fatigue, as well as emotional and social aspects [6]. Besides this, PsA has been associated with an increase in the risk of cardiovascular, endocrine/metabolic, gastrointestinal, respiratory and neuropsychiatric disorders, and uveitis [1,4].

Adequate PsA treatment is essential for the control of disease progression and its impact on the health of affected patients. Treatment comprises nonpharmacological and pharmacological measures. The so-called disease-modifying antirheumatic drugs (DMARDs) are commonly characterized by their capacity to decrease or revert signals and symptoms, functional impairment, QoL impairment, occupational disability and joint damage progression and, therefore, could interfere with the entire disease process. They are divided into three different classes: conventional synthetic DMARDs (csDMARDs), biological DMARDs (bDMARDs) and target-specific synthetic DMARDs [7].

After incorporation of DMARDs into the National Health System (Sistema Único de Saúde - SUS) of Brazil in 2009, few studies have evaluated the profile of request and use of these medications [8]. Considering this fact, the performance evaluation of technologies applied to health, which consists of the continued evaluation of incorporated technologies and the analysis of results obtained in the context of a health system, become an important tool, particularly in a scenery of successive increases in health costs, limitation of public health expenditures and significant technological advancement achieved in the last few years, which points to the need for postmarketing and/or postincorporation studies [9].

Besides this, the literature indicates that biological therapies, generally, lose their effectiveness over time [10,11]. There are few data from clinical trials regarding the comparative efficacy/effectiveness of medications for PsA treatment [10]. Finally, the long-term safety and the real purpose profile of medications could not be adequately evaluated in clinical trials, due to the restriction of such studies thanks to, in particular, inclusion and size criteria being driven by primary efficacy outcomes that result in low external validity for the real-world population [12].

In this context, the medication persistence has been interpreted as a composite measure when considering effectiveness, safety and utility in real life for rheumatic diseases. The more common reasons for the interruption of tumor necrosis factor inhibitors (anti-TNFs) were reported as effectiveness lack or loss and adverse events. However, the persistence in the use of medications could also be influenced by other factors, such as the available number of alternative treatment options and the characteristics of the population of treated patients [10,13].

Thus, the purpose of this present study is to describe, in the ambit of SUS, the profile of a sample of PsA patients in Brazil, evaluating their medication persistence, as a ‘proxy’ of both effectiveness and safety related to treatment.

## Materials & methods

This is a historical cohort of patients requiring PsA treatment in the period between 4 November 2014 and 31 December 2016 in the SUS. These patients were seen in the Belo Horizonte Health Region, which comprises a population of over five million inhabitants distributed between 39 municipalities.

The patients who requested csDMARDs or anti-TNF drugs for the treatment of PsA were identified in the Pharmaceutical Services Management System (SiGAF), where the data of requesting and authorization for use of such medications were available.

The variables analyzed at baseline were the information contained in the documentation (administrative process) extracted from the SiGAF for the request of DMARDs, namely: sociodemographic profile; patients’ clinical profile; requested medications; examinations defined in the documents listing and those necessary for opening the treatment process for PsA.

The patients who started the use of csDMARDs or anti-TNFs and had not utilized previous therapy with medication of this same class were considered eligible. The therapy administered at study start was considered: anti-TNF (adalimumab, etanercept or infliximab) and csDMARD (methotrexate or leflunomide). Patients who started treatment with sulfasalazine or cyclosporine were excluded due to the small number of requests for these medications. The date of entrance in the cohort was defined as the first date of the dispensation of the medication in the Integrated System of Hospital Management (SIGH). All the patients were followed up to the date of the final follow-up (December/2017) or death.

The outcome evaluated was persistence, defined as the time duration between the medication's first dispensation and discontinuation. Therapy discontinuation was defined as the absence of medication dispensation for more than 90 days, verified in the Integrated System of Hospital Management (SIGH). Three comparison groups were built: anti-TNF versus csDMARD (between the classes); anti-TNF (adalimumab versus etanercept and infliximab) and csDMARD (leflunomide versus methotrexate). The proportion of persistent individuals was evaluated at 6 and 12 months of follow-up and the factors associated with persistence were verified.

The independent variables considered in the analysis of factors associated with persistence were sex, age range, body mass index (BMI), C-reactive protein examination and erythrocyte sedimentation rate, medications in use, axial impairment, peripheral impairment, nail dystrophy, bone neoformation and dactylitis.

The continuous variables of interest were graphically evaluated using the Shapiro Wilk test for verification of data normality and for the definition of the measures of central tendency and dispersion. The categorical variables were described by means of distribution of frequencies and the continuous variables by means of median (interquartile range). The proportions of persistence between the groups and between the medications were compared by means of the Pearson's χ^2^ test. The average time of persistence between the groups and between the medications were compared by means of *T*-test (two comparators) or variance analysis (ANOVA) with adjustment by the method of Bonferroni (three comparators). Kaplan–Meier survival curves were elaborated to verify the time up to treatment discontinuation, that is, the loss of treatment persistence with the medications after 6 and 12 months. The log-rank test was utilized to compare the medication persistence between the study groups. The regression by the model of proportional risks of bivariate and multivariable COX was utilized to verify the variables independently associated with time up to treatment discontinuation. A significance level of 20% has been adopted for the bivariate analysis and of 5% for the multivariable analysis. The statistical analyses were conducted utilizing the software STATA version 15.1.

This study was approved by the Institutional Review Board/Independent Ethics Committee of the Federal University of Minas Gerais (UFMG), appraisals ETIC 0069.0.203.000-1 and CAAE 44121315.2.0000.5149.

## Results

**Figure 1.**
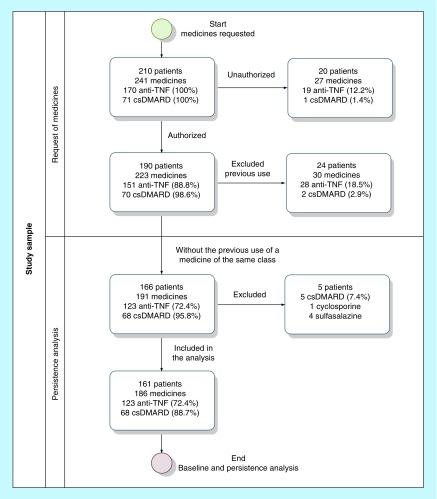
**Flowchart with the selection of patients eligible for the study.**

A total of 241 medications were requested by 210 patients, out of which 161 patients were included with the request of 186 medications (123 anti-TNF and 63 csDMARDs; Figure 1). Adalimumab (61%) was the most used anti-TNF, followed by etanercept (27.6%) and infliximab (11.4%). Among the csDMARDs, 54% of patients started methotrexate and 46% leflunomide (Table 1).

**Table 1.** **Profiles of patients who started the use of medications and were included in the persistence analysis.**

**Variables**	**Total (161)**	**%**	**Anti-TNF (123)**	**%**	**csDMARD (63)**	**%**	**p-value**
**Sex**							0.106

Female	91	56.5	64	52	40	64.5	

Male	70	43.5	59	48	22	35.5	

***Age in years***	*51.6 (42.5–59.5)*		*51.6 (41.4–59.4)*		*50.2 (43.7–58.0)*		0.966

**Race**							0.597

White	72	52.5	56	53.8	25	47.2	

Brown	61	44.5	44	42.3	27	51.0	

Black	4	3	4	3.9	1	1.9	

***BMI in kilograms***	*26.4 (23.9–29.9)*		*26.9 (23.6–29.9)*		*27.8 (25.4–30.9)*		0.045

Normal	56	36.6	48	41.4	14	23.3	0.053

Overweight	59	38.6	40	34.5	29	48.3	

Obesity	38	24.8	28	24.1	17	28.4	

**Drugs in use**							

Anti-TNF	123	66.1	123	100.0	–	–	

Adalimumab	75	40.3	75	61.0	–	–	

Etanercept	34	18.3	34	27.6	–	–	

Infliximab	14	7.5	14	11.4	–	–	

csDMARD	63	33.9	–	–	63	100.0	

Methotrexate	34	15.6	–	–	34	54.0	

Leflunomide	29	18.3	–	–	29	46.0	

**Medical specialty**							<0.001

Reumatology	124	77.0	92	74.8	52	83.9	

Dermatology	26	16.2	25	20.3	1	1.6	

Medical clinics	10	6.2	5	4.1	8	12.9	

Without specialty	1	0.6	1	0.8	1	1.6	

**CASPAR**							

Personal psoriasis	139	95.9	110	96.5	51	94.4	0.518

Family psoriasis	9	6.2	5	4.4	5	9.3	0.212

Nail dystrophy	63	46	48	44.9	28	53.8	0.287

Negative rheumatoid factor	107	73.8	84	73.7	43	79.6	0.402

Dactylitis	79	54.5	60	52.6	30	55.6	0.723

Bone neoformation	44	30.3	33	29.0	21	38.9	0.198

**Impairment**							

Axial	83	60.6	70	64.8	22	44.0	0.014

Peripheral	132	94.3	111	94.6	46	92.0	0.528

Enthesitis	46	50.0	41	52.3	10	37.0	0.147

***CRP mg/l***	*6.0 (1.8–14.8)*		*5.0 (1.5–13.5)*		*7.7 (2.4–25.4)*		0.007

>5 mg/l	83	55.7	59	51.3	38	66.7	0.056

***ESR in 60 min***	*14.0 (6.0–28.0)*		*13.0 (5.0–25.0)*		*20.0 (9.5–38.0)*		0.015

>8/10 mg (woman/man)	96	67.1	76	66.1	34	75.6	0.315

***Rheumatoid factor***	*10.0 (7.7–10.0)*		*10.0 (7.6–10.0)*		*10.0 (8.0–11.0)*		0.277

***ALT***	*20.0 (17.0–27.0)*		*20.0 (17.0–27.0)*		*21.0 (18.0–28.0)*		0.236

***AST***	*21.0 (15.0–32.0)*		*21.5 (15.0–32.0)*		*22.5 (13.0–35.0)*		0.532

Italic = Median and interquartile range.

CASPAR: Criterion of Classification of Psoriatic Arthritis; csDMARD: Conventional synthetic disease-modifying antirheumatic drug; ESR: Erythrocyte sedimentation rate.

According to the Criterion of Classification of Psoriatic Arthritis (CASPAR), 95.9% of patients presented personal psoriasis, 6.2% presented familial psoriasis, 46.0% had nail dystrophy, 73.8% had negative rheumatoid factor, 54.5% has dactylitis and 30.3% had bone neoformation. In addition, 94.3% of patients have been described as presenting peripheral impairment and 60.6% presented axial impairment. Enthesitis was observed in 50.0% of patients (enthesitis data were available only in 57.1% of patients; Table 1).

It was observed that patients using anti-TNF agents presented a higher index of axial impairment than those using csDMARDs. The higher portion of medication requests came from rheumatologists’ physicians. The baseline characteristics of patients starting use of csDMARDs and anti-TNF agents are presented in Table 1. The main differences between the patients from the two groups were in the BMI value, the specialty of the physician responsible for the prescription and in the median values of the examinations of inflammatory activity (C-reactive protein and erythrocyte sedimentation rate).

At 6 and 12 months, individuals using anti-TNF agents presented higher persistence as compared with individuals under csDMARD therapy at 6 and 12 months (χ^2^: < 0.001; *T*-test: p < 0.001). Adalimumab and etanercept presented similar persistent patients’ proportions and persistence average times. Infliximab presented lower persistent patients’ proportion and persistence average time, but without statistical significance, as compared with adalimumab and etanercept (χ^2^: > 0.05, Bonferroni: > 0.05). Leflunomide and methotrexate had close proportions and persistence average time, without any statistical difference in the class of csDMARDs (χ^2^: p > 0.05; *T*-test: p > 0.05; Table 2).

**Table 2.** **Persistence in the use of anti-TNF and conventional synthetic disease-modifying antirheumatic drug medications in individuals with psoriatic arthritis.**

**Medicine**	**% persistent 6 months**	**% persistent 12 months**	**Average in days 6 months (95% CI)**	**Average in days 12 months (95% CI)**
**Anti-TNF**	83.4	66.4	166.5 (159.6–173.3)	297.3 (278.0–316.5)

Adalimumab	86.5	69.7	170.4 (162.9–178.0)	306.1 (282.4–329.9)

Etanercept	84.4	69.0	168.5 (157.2–179.8)	306.9 (274.6–339.1)

Infliximab	64.7	43.8	140.4 (106.4–174.3)	229.9 (156.9–303.0)

**csDMARD**	50.8	35.6	135.5 (120.3–150.7)	207.2 (171.7–242.7)

Leflunomide	51.7	37.0	139.1 (116.5–161.7)	209.9 (157.8–262.0)

Methotrexate	50.0	34.4	132.4 (111.6–153.2)	204.9 (155.6–254.2)

csDMARD: Conventional synthetic disease-modifying antirheumatic drug.

**Figure 2.**
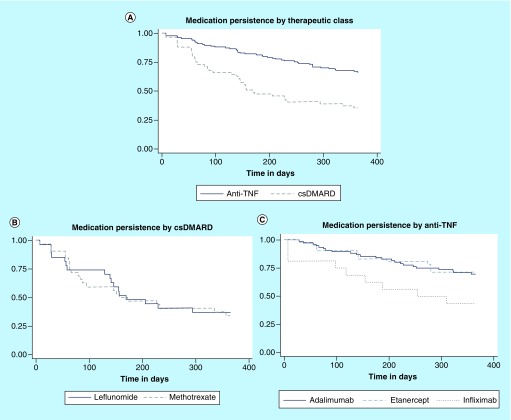
**Kaplan–Meier curves with the persistence in the treatment using anti-TNF and conventional synthetic disease-modifying antirheumatic drug.** **(A)** Medication persistence per therapeutic class. **(B)** Medication persistence per csDMARD used. **(C)** Medication persistence per anti-TNF used. csDMARD: Conventional synthetic disease-modifying antirheumatic drug.

Another observation was that individuals making use of infliximab presented lower persistence as compared with those using adalimumab and etanercept after 6 months (log-rank: p = 0.054) and 12 months (log-rank: p = 0.056). However, this observed difference restricted itself to the limit of statistical significance. In the class of csDMARDs, statistically significant difference has also not been observed in the persistence values of both leflunomide and methotrexate after 6 months (log-rank: p = 0.882) and 12 months (log-rank: p = 0.841). Individuals using anti-TNF agents presented higher persistence to treatment when compared with individuals using csDMARD (log-rank: p < 0.001; Figure 2).

In the adjusted Cox model, the factors associated with medication non-persistence with anti-TNF agents after 6 and 12 months were the female sex and use of infliximab (Table 3). Factors associated with medication non-persistence have not been found with csDMARD medications after 6 and 12 months.

**Table 3.** **Persistence predictors in the use of anti-TNF agents after 6 and 12 months.**

**Variables**	**Anti-TNF – 6 months**			

**HR crude**	**p-value**	**HR adjusted**	**p-value**	
**Sex**				

Male	1		1	

Female	2.08 (0.90–4.83)	0.087	2.42 (1.03–5.69)	**0.043**

**Anti-TNF**				

Adalimumab	1		1	

Etanercept	1.16 (0.46–2.96)	0.749	1.20 (0.48–3.07)	0.749

Infliximab	3.08 (1.15–8.20)	0.025	3.73 (1.38–10.11)	**0.010**

**Age**				

Until 50 years	1		–	–

>50 years	0.47 (0.22–1.10)	0.086	–	–

**Variables**	**Anti-TNF – 12 months**			

	**HR crude**	**p-value**	**HR adjusted**	**p-value**

**Sex**				

Male	1		1	

Female	2.23 (1.20–4.15)	0.011	2.65 (1.40–5.02)	**0.003**

**Anti-TNF**				

Adalimumab	1		1	

Etanercept	1.01 (0.52–2.01)	0.96	1.07 (0.54–2.12)	0.749

Infliximab	2.41 (1.11–5.21)	0.025	3.25 (1.47–7.21)	**0.004**

**CRP**				

Until 5 mg/l	1		–	–

>5 mg/l	0.66 (0.36–1.21)	0.177	–	–

***Rheumatoid factor***				

Negative	1		–	–

Positive	2.64 (1.03–6.77)	0.042	–	–

Only the variables with a p-value < 0.20 in the bivariate analysis were shown.

1 (reference category).

Bold: variables with significant results in the final model - multivariable analysis.

HR: Hazard ratio.

## Discussion

This is a new study conducted in Brazil, whose purpose is to evaluate PsA patients treated with anti-TNF and csDMARD medications offered by means of the SUS in a real-life scenario. The respective results have demonstrated that medication persistence was higher for patients making use of anti-TNF agents as compared with those using csDMARDs, which could be partially explained by the fact that anti-TNF agents, currently, represent the last line of treatment for PsA at SUS [14]. Other different hypotheses point to the lower acquisition price of csDMARDs, which allows their purchase by the patients themselves by means of direct disbursement and, finally, the delay in disease diagnosis due to difficult access to a rheumatologist. In the latter case, treatment starts with the disease already in a more advanced stage, resulting in less satisfactory results in terms of effective disease control [15].

Both the use of infliximab and female sex were identified as factors associated with nonpersistence to treatment with anti-TNF agents and corroborate the results from other studies [16–19]. The lower persistence for women with PsA could be explained by the higher duration of disease symptoms, higher BMI, higher count of swollen and tender joints, higher disease activity score, higher polyarticular involvement, higher alteration in the inflammatory activity test examinations, higher fatigue intensity and higher functional impairment as compared with men [20,21].

An observational study of the British Society for Rheumatology has demonstrated that the main reasons for the higher discontinuation in the use of infliximab are the lower effectiveness and higher quantity of adverse events occurring with the use of this anti-TNF agent when compared with etanercept and adalimumab [18]. In this sense, the use of infliximab presents the higher potential of immunogenicity, with the development of anti-drug antibodies (ADA), as compared with the remaining anti-TNF agents. The anti-drug antibodies (ADA) are associated with decrease of clinical response and increasing incidence of reactions to infusion as well as to reactions at the application site [22,23]. Besides this, lower adhesion to treatment with infliximab has been demonstrated in other immunomediated diseases when compared with other anti-TNF agents [24], which could be explained by its higher potential for adverse reactions, mainly infusional ones and in the application site, and by its administration path [18,22–24]; patients with rheumatic diseases might present preference for subcutaneous administration instead of intravenously and for home administration instead of hospital [24]. However, this result should be interpreted with caution as infliximab has shown better persistence in particular subsets, such as in oligoarticular-PsA [26].

Differences in the persistence to treatment have not been observed between the csDMARD medications. A study has observed a persistence of 87.7% with leflunomide after 6 months in individuals with PsA who presented the main reasons for discontinuation to be the adverse events (51%) and effectiveness loss (33%) [27]. Asiri *et al*. have found a persistence of 64.7% after 1 year of follow-up of individuals with PsA using leflunomide [28]. Curtis *et al*., in a systematic review, observed a persistence of 50–94% for methotrexate in patients with rheumatoid arthritis [29]. Such results are higher to those observed in the PsA patients participating in this present study. It should be reinforced that few studies have been addressed to the action of csDMARDs in the PsA population.

Concerning anti-TNF agents, different studies have demonstrated that persistence could vary from 61 to 79% in the first year of follow-up in different countries [17,18,30,31]. Aaltonen *et al*. observed a persistence of 90% at 6 months and 80% at 12 months of follow-up using anti-TNF agents, without differences in the persistence between the first, second and third lines of treatment [16]. The persistence after 12 months, with the use of anti-TNF agents in these studies, was somewhat higher or similar to that verified in Brazilian individuals using anti-TNF agents and participating in the present study.

There was a mild predominance of individuals of female sex and who were overweight, which was also observed in a Canadian study [31]. The mean age was similar to that of other studies [16,32].

Adalimumab was the most requested anti-TNF agent, followed by etanercept. This anti-TNF utilization profile is consistent with the literature, where adalimumab has been the medication most commonly utilized in the treatment of PsA and other rheumatic diseases in Brazil [33–35]. This could be partially explained as adalimumab presents additional treatment indications, such as uveitis, and this is a characteristic that is not shared with etanercept. Besides this, in the SUS, there are no centers of assisted therapy for the infusion of medications, which explains the lower prescription of Infliximab.

Among the csDMARDs, the medications methotrexate and leflunomide were the most requested ones. According to the European League against Rheumatism (EULAR), methotrexate should be the csDMARD of choice in patients with PsA presenting relevant cutaneous involvement [7]. Additionally, methotrexate has been the csDMARD most utilized in association with anti-TNF agents in the treatment of PsA [17,35]. Leflunomide has also been demonstrated to be an effective alternative for the peripheral and cutaneous impairment at PsA [14]. The csDMARDs were less requested when compared with anti-TNF agents. This could be explained in part by the fact that csDMARDs are preferentially utilized in patients with peripheral impairment, due to low proven efficacy in the axial impairment [7,14].

With respect to disease classification by the Criterion of Classification of Psoriatic Arthritis (CASPAR), the occurrence was verified of a predominance of peripheral involvement, with personal psoriasis in the large majority of cases and predominance of negative rheumatoid factor, which corresponds to the findings of Taylor *et al*. [36].

The higher frequency of requests from rheumatologist physicians is in concordance with the PsA treatment protocol in Brazil. This protocol recommends PsA individuals are met by a team, in a specialized service, counting on a rheumatologist for the appropriate diagnosis, inclusion in the treatment and follow-up [14]. The prescription by different medical specialties (rheumatology, dermatology and general practice) could be explained by the fact that psoriasis diagnosis precedes the one of arthritis in 75% of cases [37]. Furthermore, the general practice is a medical residency that precedes the formation in rheumatology in Brazil.

Recommendations and guidelines highlight that a delayed diagnosis is a great challenge that needs to be overcome, as it negatively impacts the treatment result, and strategies promoting the early forwarding of patients and decreasing the delay in diagnosis and treatment of inflammatory arthritis have been proposed [38]. This is a problem that has been confronted in Brazil and the challenges faced are in the concentration of rheumatologist physicians in the larger cities. There are difficulties in the early forwarding of patients to a rheumatologist by the primary care physicians, mainly for patients from the countryside and small cities, as well as in the follow-up maintenance and treatment management of patients after that forwarding [39].

Another alternative to improve health outcomes is the implementation of pharmaceutical care in the SUS. Recent results demonstrate that this alternative is cost-effective and improves the quality of healthcare. [40,41]. Thus, SUS would be better able to provide an integral care, which is one of its basic premises. In this sense, complementary measures could be useful for the maximization of results and to qualify the use of medications; such measures, for instance, could include the improvement of access to rheumatologist physicians, the implementation of more efficient treatment management tools and the integration of a multidisciplinary team to follow-up PsA patients.

The use of administrative data possesses important limitations. One of them is that the objective of the records is for authorization/reimbursement/dispensation of medications from the health system and not for data collection for clinical–epidemiological studies. Therefore, this study does not reflect direct clinical effectiveness but provides a better understanding of the occurrence of nonpersistence in the PsA patients served at SUS. Another important limitation is the lack of data concerning the administrative processes, such as clinical measures, time of disease duration and mandatory examinations, which could give more information about the patients seen. The nonavailability of csDMARDs for dispensation in given periods could have negatively influenced the persistence of such medications.

Despite these limitations, the study presents potentialities. The csDMARD and anti-TNF agents were included at SUS in 2009 and were not the target of previous evaluations in the Brazilian context. Our results demonstrate the persistence in the use of these medications in a real life scenario, making it the first postincorporation study addressed to PsA in Brazil with this focus. In addition, the study has identified relevant aspects that effectively influence the treatment of PsA at SUS. The knowledge about the utilization of these medications in the Brazilian context is relevant, as the majority of publications reflect the reality of populations of other different countries. In this sense, it is observed the lower persistence to biological treatment by women, which requires a closer follow-up and an approach of the specific needs in this group of patients to improve the response to treatment and, consequently, the results in health. Finally, few studies have proposed to evaluate the use of csDMARDs within a real-life scenario. Thus, it is necessary to investigate if the persistence in the use of these medications has decreased over time, mainly in a context of innovation of medications and consequent increase in the number of alternative therapies for PsA.

## Conclusion

The results demonstrate that anti-TNF agents are important therapeutic alternatives and present lower discontinuation as compared with csDMARDs after 6 and 12 months of follow-up. Adalimumab and etanercept presented similar persistence results in anti-TNF group and leflunomide and methotrexate in the csDMARDs group. Medication persistence was lower in Brazil than in other countries. Additional measures are important to improve outcomes in medication persistence such as increasing access to rheumatologists and implementing a pharmaceutical care strategy.

## Future perspective

In Brazil, the Pharmaceutical Services is the main tool to get access to medicine in the SUS. However, anti-TNF and csDMARD drugs have not been previously evaluated for their efficacy, safety and cost-effectiveness for PsA in Brazil. In a time of economic recession, we need to establish a useful tool for decision-making and to identify policy options for containment and reordering of costs. In addition, as the volume, complexity and cost of new medical technologies increase, the need to assess benefits, risks and costs become increasingly important, both for new technologies and those already incorporated into the health system but not previously evaluated. In this context, the analysis of health performance is useful to subsidize the renegotiation of health prices and the optimization of the application of financial resources, with the purpose of contributing to the efficiency of public management and, consequently, to the pharmaceutical services and SUS.

**Summary points**

- The conventional synthetic disease-modifying antirheumatic drug (csDMARD) and TNF inhibitor (anti-TNF) agents were included at National Health System (SUS) in 2009 and were not subject to previous evaluations in the Brazilian context.
- The medication persistence for psoriatic arthritis varies among different countries and data for developing countries were not found.
- A total of 161 psoriatic arthritis patients were included in the present study and 186 treatments were evaluated.
- The patients under anti-TNF treatment presented higher persistence as compared with patients under csDMARDs treatments at 6 and 12 months.
- Adalimumab and etanercept presented similar persistence results in the anti-TNF group.
- Leflunomide and methotrexate presented similar persistence results in the group of csDMARDs.
- The factors associated with nonpersistence with anti-TNF agents were female sex and use of infliximab.
- Factors associated with nonpersistence with csDMARDs were not found.
- These results are very important because the the majority of publications reflect the reality of populations in other different countries.
